# Mechanism of human nail poration by high-repetition-rate, femtosecond laser ablation

**DOI:** 10.1007/s13346-019-00638-x

**Published:** 2019-04-23

**Authors:** Simon Vanstone, James M. Stone, Sergey N. Gordeev, Richard H. Guy

**Affiliations:** 1 Department of Physics, University of Bath, Claverton Down, Bath, BA2 7AY UK; 2 Department of Pharmacy & Pharmacology, University of Bath, Claverton Down, Bath, BA2 7AY UK; 3 Centre for Nanoscience & Nanotechnology, University of Bath, Claverton Down, Bath, BA2 7AY UK; 4 Centre for Therapeutic Innovation and Centre for Biosensors, Bioelectronics & Biodevices, University of Bath, Claverton Down, Bath, BA2 7AY UK

**Keywords:** Human nail, Femtosecond pulsed laser ablation, Nail poration, Fluorescence microscopy, Thermal mapping, Drug delivery

## Abstract

Optical poration, or drilling, of the human nail has the potential to drastically improve transungual drug delivery. However, this approach is accompanied by thermal damage to the nail tissue surrounding the laser radiation-created pore. In this paper, fluorescence microscopy has been employed to quantitatively evaluate thermal damage to the nail induced by laser ablation with 80 MHz, nanojoule, femtosecond pulses delivered via a hollow-core fibre. An empirical relation has been established between the intensity of the resulting fluorescence signal and temperature to which the nail was exposed. Using this relationship, detailed temperature maps have been created of the areas surrounding the pores, enabling the mechanism of poration to be better understood. It was deduced that plasma-mediated ablation is primarily responsible for nail tissue ablation at the centre of the pore, while cumulative photothermal processes dominate at the pore edges. It is concluded, furthermore, that temperature mapping represents a useful new tool with which to optimise the process of nail poration. The method is potentially generic and may be applicable to other biological materials.

## Introduction

Lasers have been used for medical purposes since 1961 when Leon Goldman employed a ruby laser to remove tattoos and other pigmented lesions from the skin [1]. Common laser applications are removal of malignant tissue [2], making precise cuts in soft and hard tissues [3], and the optical drilling of holes in bone, nail and skin [4, 5]. A typical side effect associated with such treatments is substantial thermal damage to the surrounding tissue caused by heat-induced molecular changes in proteins [6], and this has become a barrier to the wider implementation of lasers in medicine.

Unwanted thermal damage can be reduced by shortening the time of interaction of the laser beam with the tissue through the use of ultrashort pulses. The energy for efficient ablation decreases with decreasing pulse duration from 0.25–250 μJ for nanosecond pulses [7] to a few nanojoules for femtosecond pulses [8]. This observation was applied [4] to show that laser ablation with 350-fs laser pulses (*λ* = 1.05 μm) of hard tissues, such as nail, mid-ear bone, dentin and enamel, generated very little thermal damage. To achieve an effective removal rate, a short-pulse laser system should operate at a high repetition rate and this is now possible with the development of ultrafast laser systems producing femtosecond pulses with repetition rates in the MHz range. However, as typical thermal relaxation times in biological tissues are on the order of milliseconds, heat nonetheless accumulates in the tissue over time for repetition rates > 1 kHz. Computer simulation shows that other cumulative effects may also be operative, causing the ablation mechanism to change [7] and resulting in significant heat accumulation and potentially serious thermal damage. The latter, though, can be reduced by optimising the laser ablation regime, i.e. by tuning the radiation wavelength, the amplitude of the pulses and their repetition rate.

An area of particular interest is the laser poration of human skin to enhance (trans)dermal drug delivery. Early research showed that the efficiency of transport through laser-created pores was variable and sensitively dependent on the laser radiation regime used [9]. For continuous radiation and long-pulse (~ 10^−3^ s) regimes, the pores are produced via photothermal ablation of skin tissue and are accompanied by thermal damage around the pore and tissue coagulation at the edges, sealing off the pore and drastically reducing the efficiency of drug diffusion [10]. In contrast, for ultrashort pulses (pico-/femtosecond range), the light energy is temporally and spatially confined. Each pulse evaporates a small volume of tissue, turning it into plasma. This causes a microexplosion that expels the evaporated material from the pore without significant thermal damage to the surrounding tissue. The shorter the pulse duration, the less the thermal damage. It was recently demonstrated that laser poration of mammalian skin with femtosecond pulses produces pores with little thermal damage and enables a substantial enhancement of drug permeation [11].

The treatment of nail disease is more challenging. The nail plate, typically 0.2–0.5 mm thick, is an effective barrier of ~ 100 layers of tightly bound, dead, keratinized cells. Current topical treatment methods include drug-containing lacquers, solutions and gels that have extremely low efficacy [12]. Nail diseases are increasingly prevalent with the most common, onychomycosis, affecting 10–40% of the population [13] and the treatment of which has already generated an annual, global market of nearly $3 billion today (https://www.persistencemarketresearch.com/market-research/dermatophytic-onychomycosis-therapeutics-market.asp, https://www.coherentmarketinsights.com/market-insight/dermatophytic-onychomycosis-treatment-market-2053). Oral antifungals are also commonly used, but multi-month treatment durations and high recurrence rates (as high as 50% [14]) result in poor patient compliance. Further, oral antifungal therapy is not indicated for diabetics and the elderly, due to significant side effects and incompatibility with other medicines [15]. Several device-based techniques have been investigated for their ability to disrupt the nail barrier, including iontophoresis [16], low-frequency ultrasound [17], mechanical microporation [18] and laser poration. The first laser poration study on nail used pulse lengths of 350 fs, 15 ns and 250 μs, the shortest of which created pores with minimal collateral damage [19]. More recently, nails were successfully porated using a CO_2_ laser with a long, 3.5-ms pulse duration [20] and 92% of patients, who were treated with a topical antifungal cream post-poration, showed a beneficial response (albeit with reports of mild pain probably caused by heating of the nail and the tissue below) [21].

To enhance ablation while reducing damage to the surrounding tissue (and avoiding coagulation of tissue around the pore that may impair diffusion of a subsequently applied drug) is pretreatment with an ink that strongly absorbs light at the laser’s wavelength. This decreases the ablation power threshold on both enamel and skin [5, 22]. However, it is difficult to compare the thermal damage produced by different laser systems because no quantitative method is presently available to do so. One potential solution for biological applications is fluorescence spectroscopy [23–25]. This is because the fluorescence of biological tissue changes appreciably upon heating, e.g. when skin temperature is raised above 100 °C, the signal intensity markedly increases [5], due to denaturation of cellular proteins and collagen [26]. Therefore, the florescence intensity may provide a measure of thermal damage to the tissue. Moreover, the temperature to which the tissue sample has been exposed can be determined if an appropriate calibration curve has been established. The human nail is a logical tissue with which to examine this hypothesis due to its relatively uniform composition across its thickness [27, 28].

The research in this paper builds on previous studies, which probed the manner and extent to which femtosecond laser pulses could significantly enhance drug delivery across the skin [5] and nail [11], and describes the development of a technique to measure thermal damage to the nail caused by laser ablation. Heat-treated nails exhibit strong fluorescence, the intensity of which increases with temperature and can be used, therefore, to quantify thermal damage during laser poration. Raman microspectroscopy has been employed to acquire high-resolution, two-dimensional “thermal” maps of the areas surrounding the pore and these data can then be used to optimise the laser poration procedure: specifically, to find the optimal amplitude of laser pulses, and the repetition rate, that produces minimal thermal damage to the nail. A hollow-core negative curvature fibre (HC-NCF) was used to deliver 80-MHz femtosecond laser pulses (532-nm wavelength) to the nail, because standard solid-core fibres do not support propagation of ultrashort pulses [29]. This proved effective in facilitating reliable and reproducible poration with laser pulses of a very low energy (in the nanojoule range).

## Materials and methods

### Nail preparation

Nail clippings were collected from seven healthy adult volunteers from whom informed consent was obtained. The University of Bath Research Ethics Approval Committee for Health (EP 15/16 71) granted ethical approval for nail sampling.

**Fig. 1:**
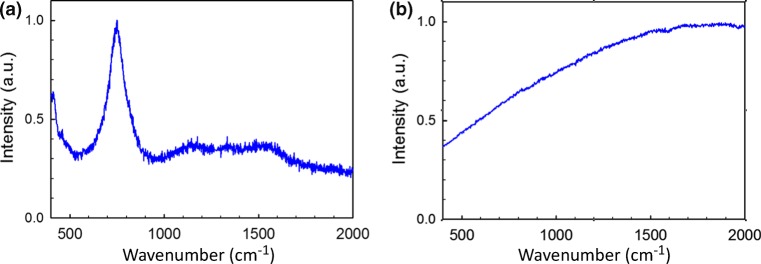
**a** Raman spectrum of Ruthenium Red. **b** Fluorescence spectrum of a nail sample that has been exposed to Ruthenium Red

The nails were cleaned with cold water before being stored in zip-lock bags at − 20 °C until use. Upon removal from the freezer, the clippings were soaked in deionised water for 30 min to rehydrate. As no poration was observed on clean samples exposed to the maximum output power of the laser system used, the nails examined in the laser poration experiments were impregnated with a dye by soaking them for 15 min in a 1 mg/mL aqueous solution of Ruthenium Red (Sigma-Aldrich, Dorset, UK). This dye absorbs strongly at the laser output wavelength, drastically reducing the poration threshold. Conveniently, Ruthenium Red has a distinct Raman peak at 750 cm^−1^ (see Fig. 1a) that, in principle, can be used to identify its presence. However, fluorescence from the nail itself is actually much stronger than the Raman signal from the dye, and the 750 cm^−1^ peak is not, in fact, detectable in dye-treated samples (see Fig. 1b); exposure to Ruthenium Red did not therefore introduce any noticeable changes in the measured fluorescence spectrum of the nail. For the temperature calibration experiments, undyed nail samples were cut into pieces roughly 1 mm^2^ in area and placed in contact with a metal plate inside a furnace. The temperature of the metal plate was monitored with two thermocouples; eight temperatures and five contact times were examined for each of three nails obtained from different donors.

### Raman spectroscopy

**Fig 2:**
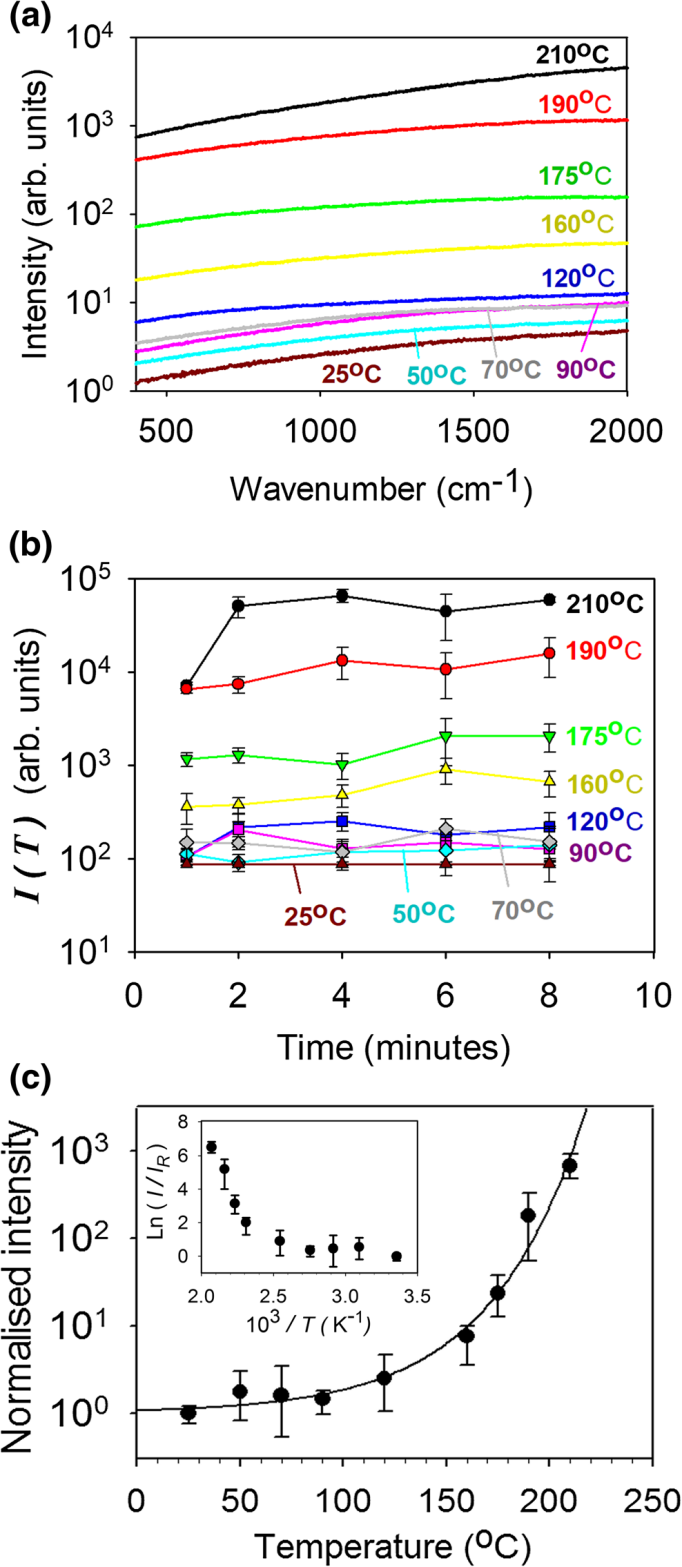
**a** Representative fluorescence spectra recorded from a nail sample after heating for 4 min as a function of temperature. All curves are normalised by the intensity of the fluorescence signal at 400 cm^−1^ at 25 °C. **b** Integrated fluorescence intensity measured from nail samples after heat treatment as functions of temperature and time. Data points represent the mean ± SD values of four spectra from each of three nail samples from different donors. **c** Normalised integrated fluorescence intensity as a function of temperature. Data points again represent the mean ± SD values of four spectra from each of three nail samples from different donors. The empirical curve fitted to the data is given by Eq. (1). The inset shows an Arrhenius transformation of the data

A Raman microscope (inVia, Renishaw, Gloucestershire, UK), equipped with a × 20 objective, was used to record the nail spectra. In preliminary experiments, infrared spectra of nails at two different excitation wavelengths 532 and 785 nm were obtained. At 785 nm, the broadband fluorescence signal is weak and typical Raman spectra with multiple peaks from various functional groups were easily detectable, in agreement with previous reports [30]. In contrast, at 532 nm, the spectrum is completely dominated by an essentially featureless fluorescence response over the entire temperature range considered (see Fig. 2a). It was found that the magnitude of this broadband signal is much more sensitive to thermal treatments than the intensity of Raman peaks corresponding to the 785-nm excitation, so only data for the excitation wavelength of 532 nm are reported below. Four replicate measurements were taken from each sample in the temperature calibration experiments.

### Laser poration of human nails

A ytterbium-doped fibre laser (Fianium FP-1060-5-FS, Southampton, UK) was used to porate the nails. The laser produced 5 W of average power, with 200-fs pulses at an 80-MHz repetition rate. The laser pulses were directed through a half-way plate and polarising beam splitter to control average power without changing the pulse characteristics. The beam then passed through a frequency doubling crystal (lithium triborate) to provide a wavelength of 532 nm. A mechanical shutter controlled the exposure time. As the laser had a fixed repetition rate, an optical chopper was introduced into the setup to investigate how the grouping of pulses affected the degree of thermal damage. The chopper disc had 10 open/closed windows in a 1:1 ratio. When set to a frequency of 20 Hz, groups of pulses lasting 2.5 ms were obtained, separating identical periods with no pulses, during which any accumulated heat could dissipate. The laser pulses were delivered to the nail via a HC-NCF, which was accurately positioned using a mechanical stage. The diameter of the light spot on the nail surface was typically ~ 20 μm. Table 1 shows the regimens used for poration and the geometrical parameters of the pores produced. The average power was measured after the light passed through the optical fibre. The ablation threshold for dyed nails was typically 40 mW. For regimens #1 and #2, the nails were fully porated; as a result, the thickness of the nail equalled the pore depth in these cases. Pore depths and diameters were measured from five locations on each sample.

**Table 1:** Light exposure protocols and pore characteristics obtained for nails from three volunteers (A, B, C). The highlighted pore depth values correspond to the nail thickness of fully porated nails (regimens #1 and #2)

Energy per pore (J)	Regimen	Pulse energy (10^−9^ J)^a^	Average power (mW)	Time (s)	Pore depths (μm)			Pore diameters (μm)		
					A	B	C	A	B	C
0.25	#1^b^	13	1000	0.25	430	370	500	120	220	100
0.084	#2	16	1300	0.063	320	292	350	105	100	90
0.063	#3	6	500	0.125	180	240	90	80	115	45
0.043	#4	16	1300	0.033	120	120	110	60	100	105
0.033	#5	13	1000	0.033	120	120	120	70	60	70
0.031	#6	6	500	0.063	100	150	100	70	80	35
0.031	#7	3	250	0.125	80	90	80	50	40	40

^a^Pulse energy was measured with a thermal power meter as the average power incident on the sample divided by the laser repetition rate

^b^Regimen #1 employed no optical chopper; all other regimens used an optical chopper producing 2.5-ms pulse trains

Regimens #2 and #4 employed the maximum pulse energy that was achievable with the laser used. Regimen #2 produced full-depth pores, while #4 drilled through ~ 30% of the nail thickness. Regimen #1 provided full-depth pores without using the chopper; regimen #5 used pulses of the same energy for a much shorter time; regimens #3 and #6 used laser pulses of approximately 50% energy (compared to regimens #1 and #5). In regimen #7, the energy was further reduced to produce pores with minimal thermal damage. Exposure times were selected to give pores of a reasonable depth, and such that they were convenient for thermal mapping.

### Microtoming

To examine the interior of the pores, nail samples were sliced using a Reichert-Jung Ultracut E ultramicrotome (Nussloch, Germany). Although microtoming of biological samples usually requires fixing in wax or resin, the hardness of the nail rendered this step unnecessary. To prepare nail samples for microtoming, they were first trimmed before being fixed with a small dab of superglue (Loctite, Westlake, OH) in a slot in the sample holder.

## Results and discussion

### Evaluation of thermal damage at the nail surface

To determine the fluorescence intensity response as a function of temperature to which nails were exposed, samples were placed on a copper block inside a furnace for a period of between 1 and 8 min. After exposure, fluorescence spectra were acquired from four different locations on each nail. Figure 2a shows the fluorescence spectra of a clean (undyed) nail sample from a single donor after a 4-min heat exposure as a function of temperature. The intensity of the detected signal rapidly increased with temperature over the whole spectral range. Because of the strong background signal, no distinct spectral features were observed allowing the fluorescence intensity data to be correlated with the temperature to which the nail was exposed. Although, in theory, any wavenumber, *ν*, in the studied range (400–2000 cm^−1^) could be used, the average intensity over the range of Δ*v =* 700–1700 cm^−1^, calculated as *I*(*T*)$={\int}_{700}^{1700}I\left(T,v\right) d\nu /\Delta  \nu$, was selected to quantify the response at each temperature (*T*). Figure 2b shows that the calculated *I*(*T*) values are essentially independent of the time of heat treatment, the initial rise between 1 and 2 min being most probably associated with thermal equilibration of nail with the furnace temperature.

Figure 2c displays the dependence of normalised *I*(*T*) on temperature (the inset showing an Arrhenius transformation of the data). The normalisation procedure involved the following steps: fluorescence spectra were acquired from nail samples from each of the three donors as a function of temperature and the average fluorescence response, *I*(*T*), from each sample, at each temperature, was determined (in the range 700–1700 cm^−1^). These *I*(*T*) values were then normalised, for each sample, by the corresponding response at room temperature (*I*_*R*_). Finally, the averages (± SD) were calculated. The results indicate that heat treatment at temperatures below 90 °C did not have any significant effect on the normalised *I*(*T*). However, above 120 °C, *I*(*T*) increased rapidly, reaching values that were 1000-fold higher at 210 °C than those at room temperature. The sharp increment between 120 and 160 °C is possibly associated with the melting of crystalline α-keratin (reportedly at 142 °C [6, 31]), the structural and chemical disruption of which should result in a strong uplift of the fluorescence signal. Samples heated to 160 °C and above showed visible darkening and their surfaces were roughened.

Thermal damage to biological materials is usually analysed within the framework of a model [32] that treats heat damage as a thermally activated chemical process. Denaturation of proteins begins when the increase in temperature elevates the kinetic energy of the constituent molecules such that they overcome the weak hydrogen bonds and van der Waals interactions that are responsible for stability [33]. The rate of ‘damage’, expressed by the parameter Ω, is given by the so-called heat damage equation [33]

1 $$ d\varOmega / dt=P\cdotp \mathit{\exp}\left(-E/ kt\right) $$

where *P* is a frequency factor, *E* is the activation energy for denaturation, *T* is the absolute temperature, and *k* is Boltzmann’s constant.

While this model reasonably describes heat-induced damage processes at moderate temperatures (≤ 70 °C) [34], it fails at higher [35]. To compare the experimental fluorescence data with this model, the intensity of the featureless background signal (*I*) was assumed to be proportional to the damage parameter (Ω). The results in Fig. 2b, c cannot be described by Eq. (1) which predicts, for a fixed temperature, that the amount of thermal damage should be proportional to the exposure time. In contrast, the data in Fig. 2b show very little, if any, increase in the fluorescence response with treatment time and indicate, at least for higher temperatures (120–210 °C), that thermal damage saturates very quickly (for *T* < 120 °C, the measurement accuracy is too low to draw any definitive conclusion). Furthermore, the very steep increase in intensity with temperature shown in Fig. 2c cannot be described by a single process with a constant activation energy across the whole temperature range. The inset to Fig. 2c is an Arrhenius-type representation of the results, i.e. a semi-logarithmic graph of normalised *I* vs 1/*T* that exposes a highly non-linear dependence.

In principle, the data in Fig. 2c could be fitted to Eq. (1) using a temperature-dependent activation energy. However, as this would require the fitting of at least six parameters, it was decided to model the results with an empirical relation and only two fitting parameters:

2 $$ \log \left[\frac{I(T)}{I_R}\right]=\alpha \cdot \exp \left[\beta \left(T-{T}_0\right)\right], $$

where *I*(*T*)/*I*_R_ is the intensity (normalised to the room-temperature value) after heat treatment at absolute temperature *T*, *α* = 0.031 and *β* = 0.0217 K^−1^ are fitted constants, and *T*_0_ = 273 K. It follows that this equation can be used to determine, from the fluorescence-assessed *I*(*T*) post-treatment, the temperature achieved in a nail sample when exposed to laser irradiation, and to quantify any thermal damage to the surrounding tissue following a poration event. It should be noted that this equation applies when *I*(*T*) is measured at the nail surface. To determine the amount of thermal damage within the nail, the variation of the intrinsic fluorescence signal (i.e. not caused by the thermal treatment) must be taken into account as discussed below.

### Laser poration of the nail

Recent developments in Raman/fluorescence microspectroscopy now permit measurements to be performed with a spatial resolution as small as 0.5 μm. It is therefore possible to generate high-resolution maps of laser-porated nails to better understand the pore ‘drilling’ process and to identify regimens that produce minimal thermal damage, thereby ensuring the ability for safe and effective drug delivery.

Here, human nail samples were porated with a femtosecond pulsed laser. The ultrashort pulses involved can be difficult to deliver to the required location and the use of an optical fibre offers a convenient, flexible and precise solution for this challenge. However, because typical (glass core) fibres have a large optical dispersion, they are unsuitable for transporting ultrashort pulses. To circumvent this problem, therefore, low dispersion HC-NCF (designed and fabricated at the University of Bath) were used [5, 29].

The average light power (and hence the amplitude of the laser pulses) and the time of exposure were systematically examined to identify regimens that produced pores with minimal thermal damage to the surrounding tissue. Because the laser system used did not allow pulse duration or repetition rate to be varied, an optical chopper was employed to allow more time for thermal dissipation between groups of laser pulses. Table 1 summarises the different light exposure settings investigated and the diameter and depth of the pores produced. All of these pores were smaller in diameter than those reported in the literature, with less visible thermal damage surrounding them [20, 21]. Unsurprisingly, the pore depth (and, in most cases, the diameter too) increased with increasing total energy supplied.

**Fig 3:**
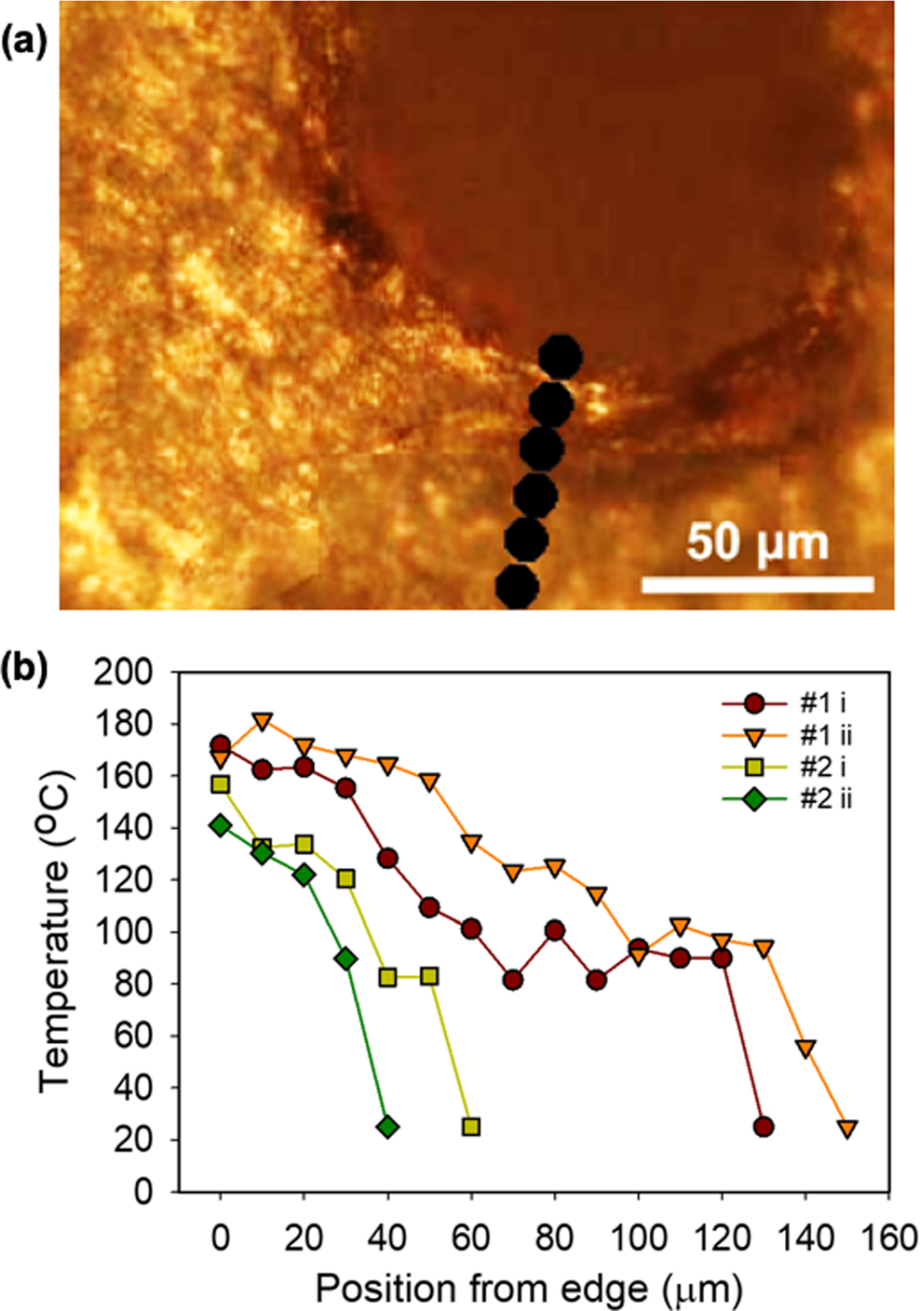
**a** Optical image of a pore created with regimen #2, and markers showing where fluorescence measurements were taken. **b** Deduced temperature profiles (from the measured *I*(*T*) values and Eq. (1)) in the vicinity of four pores created with regimens #1 and #2. The pore labelled 2ii corresponds to the image in **a**

To obtain a detailed and quantitative assessment of thermal damage, a Raman microscope was used to measure the local fluorescence spectra, *I*(*v*), at different distances from the pore edge. Figure 3a shows an example of a pore created with regimen #2 on a nail sample from volunteer B. Figure 3b shows deduced temperature profiles (from the measured *I* values and Eq. (2)) in the vicinity of four pores created with regimens #1 and #2. In each case, fluorescence spectra were taken at increments of 10 μm from the pore edge. Regimen #1 caused noticeably more thermal damage than regimen #2. In the former, the temperature reached 170–180 °C at the pore edge, decreasing to below 80 °C at a distance of 120–130 μm; in contrast, for the latter, the temperature at the pore edge was only 135–140 °C, falling to below 80 °C at 30–50 μm.

### Evaluation of thermal damage within the nail

**Fig. 4:**
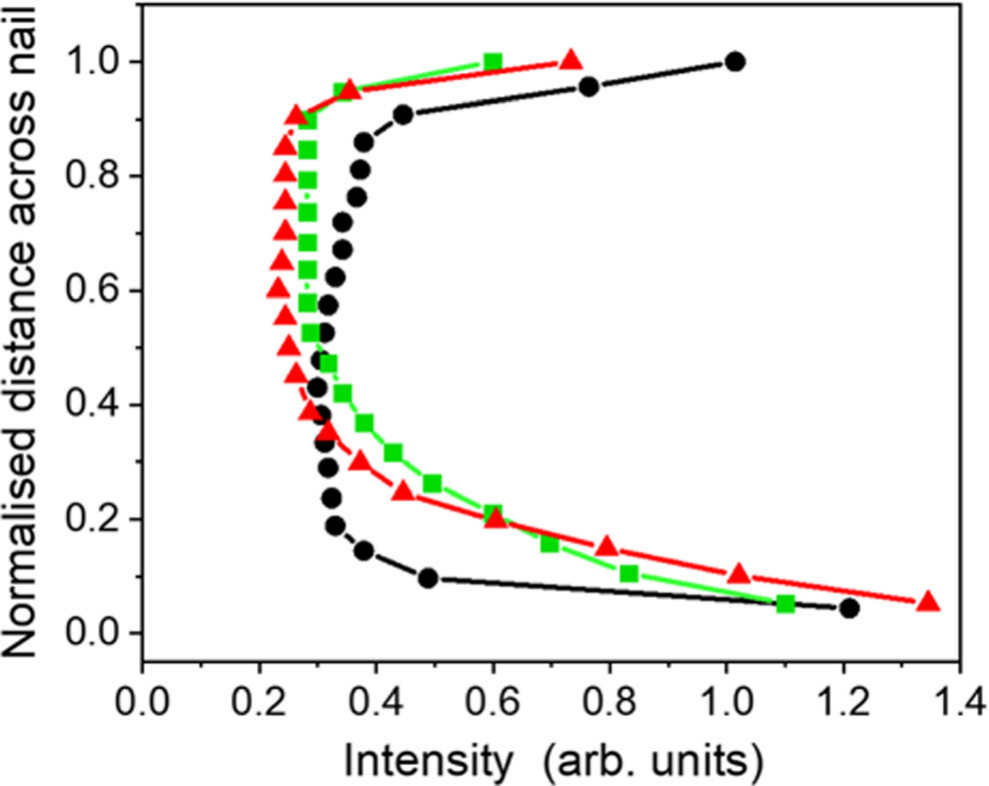
Profiles of spectrally averaged fluorescence intensity across undyed nail samples from three different donors as a function of normalised distance into the nail (0 represents the interior surface, 1 the dorsal surface)

Before measuring the thermal damage cross-sectionally around a pore, fluorescence measurements were performed on intact (non-porated and undyed) nails. Figure 4 presents the measured intensities (*I*) across nail samples from three different donors plotted as a function of normalised distance into the nail (to facilitate comparison between nails of different absolute thickness). The fluorescence response was an order of magnitude stronger near the nail edges (and greater at the ventral surface), an observation probably related to the chemical composition and physical structuring of the cells in these regions [13, 36]. It can readily be deduced that, for a nail exposed to a thermal treatment, the local fluorescence intensity depends on both temperature and depth as shown in the curves in Fig. 4.

**Fig. 5:**
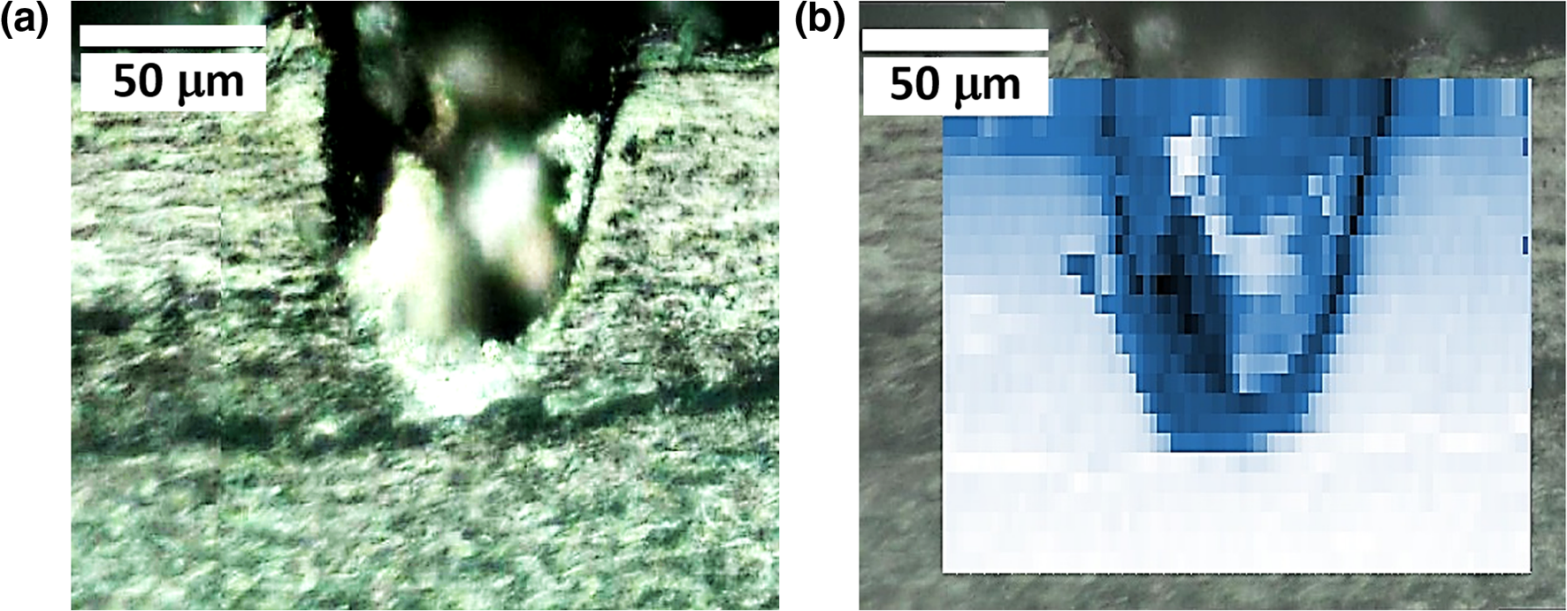
**a** Optical cross-sectional image of a partially porated nail sample from donor C porated with regimen #5. **b** The image in **a** with a superimposed fluorescence map where deeper shades reflect higher signals

After laser poration, the thermal damage around the created pores within the nail was assessed. The treated nail samples were microtomed to the point at which a cross section of a pore was revealed. Fluorescence spectra were then acquired from different locations around the pore and the corresponding *I*(*T*,*z*) values were calculated. Figure 5a is an optical image of a partially porated nail sample from donor C, while Fig. 5b shows the same image with a superimposed fluorescence map. Here, deeper shades reflect higher signals and these are found at the pore edges (as anticipated from Fig. 4). Fluorescence information from the centre of the pore (i.e. between the edges) cannot be interpreted with confidence because here the microscope was out of focus.

**Fig. 6:**
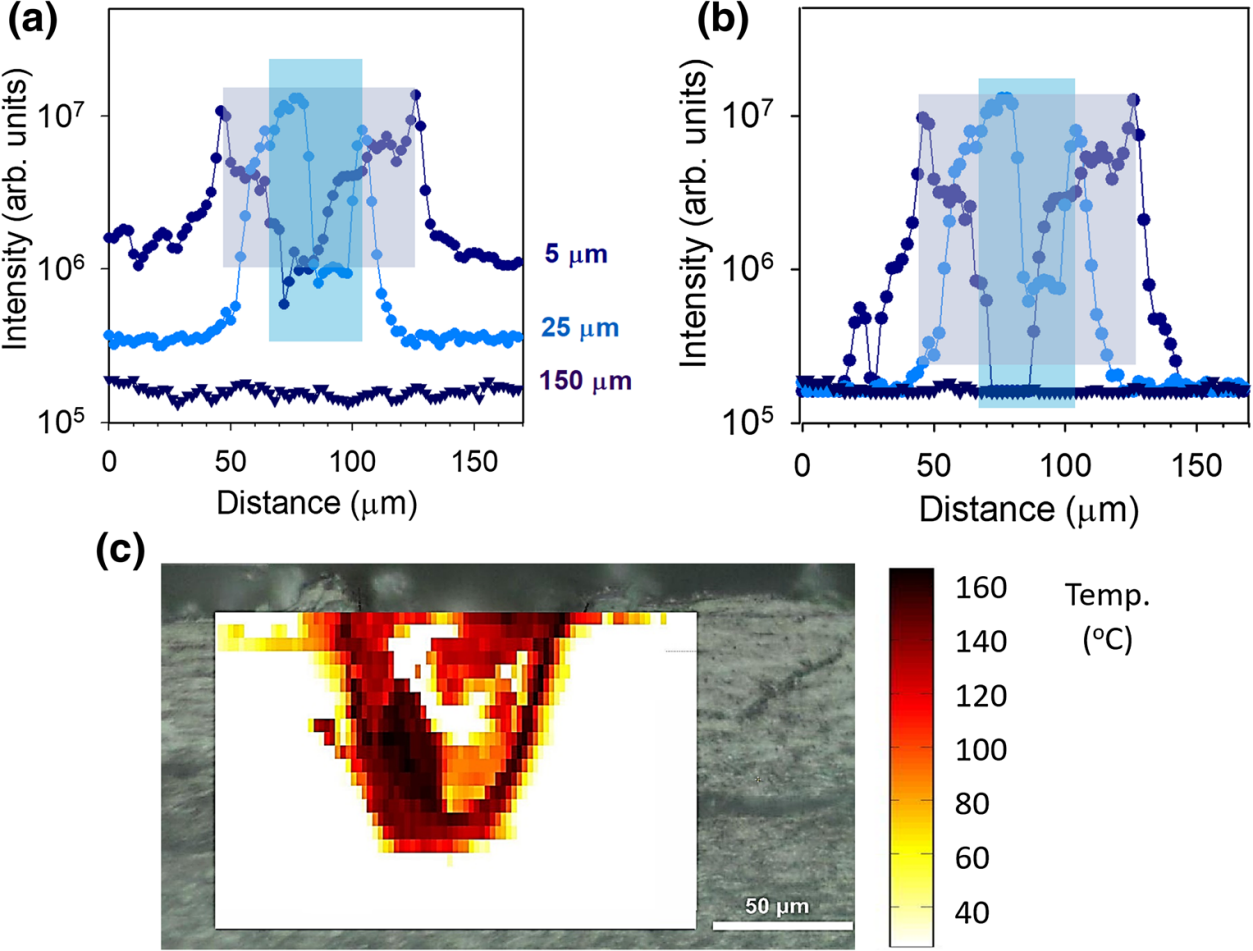
**a** Spectrally averaged fluorescence response measured across the porated sample shown in Fig. 5 at depths of 5, 25 and 150 μm into the nail. The shaded regions highlight the central regions of the pores where the measurements were out of focus (grey at 5 μm depth, light blue at 25 μm). **b** The three profiles in **a** corrected for the corresponding intensities from undamaged regions of the nail. **c** Fluorescence intensity data in **b** converted into a temperature map of thermal damage around the pore using Eq. (3)

In Fig. 6a, the fluorescence intensity at room temperature across the porated sample (Fig. 5a) is shown at depths of 5, 25 and 150 μm into the nail. The maximum intensities corresponding to the pore edges are identical at 5 and 25 μm into the nail; in contrast, the intensity from undamaged nail deeper into the sample is significantly lower. However, when the three profiles at different depths are corrected for the corresponding intensities from undamaged regions of the nail (i.e. [*I*(*T*,*z*) − *I*(*T*_R_,*z*)]), a consistent pattern of behaviour is observed (Fig. 6b). It follows that *I*(*T*,*z*) represents the sum of two terms, one dependent only on temperature, the other only on *z*, i.e. *I*(*T*,*z*) = *I*(*T*,*0*) + [*I*(*T*_*R*_,*z*) − *I*(*T*_R_,0)], where *z* = 0 corresponds to the nail surface. The subtraction of *I*(*T*_R_,0) ensures that, at the nail surface, *I*(*T*,*z*) = *I*(*T*,*0*), where the temperature dependence of *I*(*T*,*0*) is as shown in Fig. 2c. Hence, Eq. (1), which describes empirically the impact of temperature on *I*(*T*,*z*) at *z* = 0, can be generalised to any depth as follows:

3 $$ \log \left[\frac{I\left(T,z\right)-I\left({T}_R,z\right)}{I\left({T}_R,0\right)}+1\right]=\alpha \cdot \exp \left[\beta \left(T-{T}_0\right)\right] $$

This relationship can now be used to convert the fluorescence intensity data (Fig. 5b) into a temperature map of thermal damage around the laser-created pore as shown in Fig. 6c.

### Temperature maps for thermal damage in porated nails

**Fig. 7:**
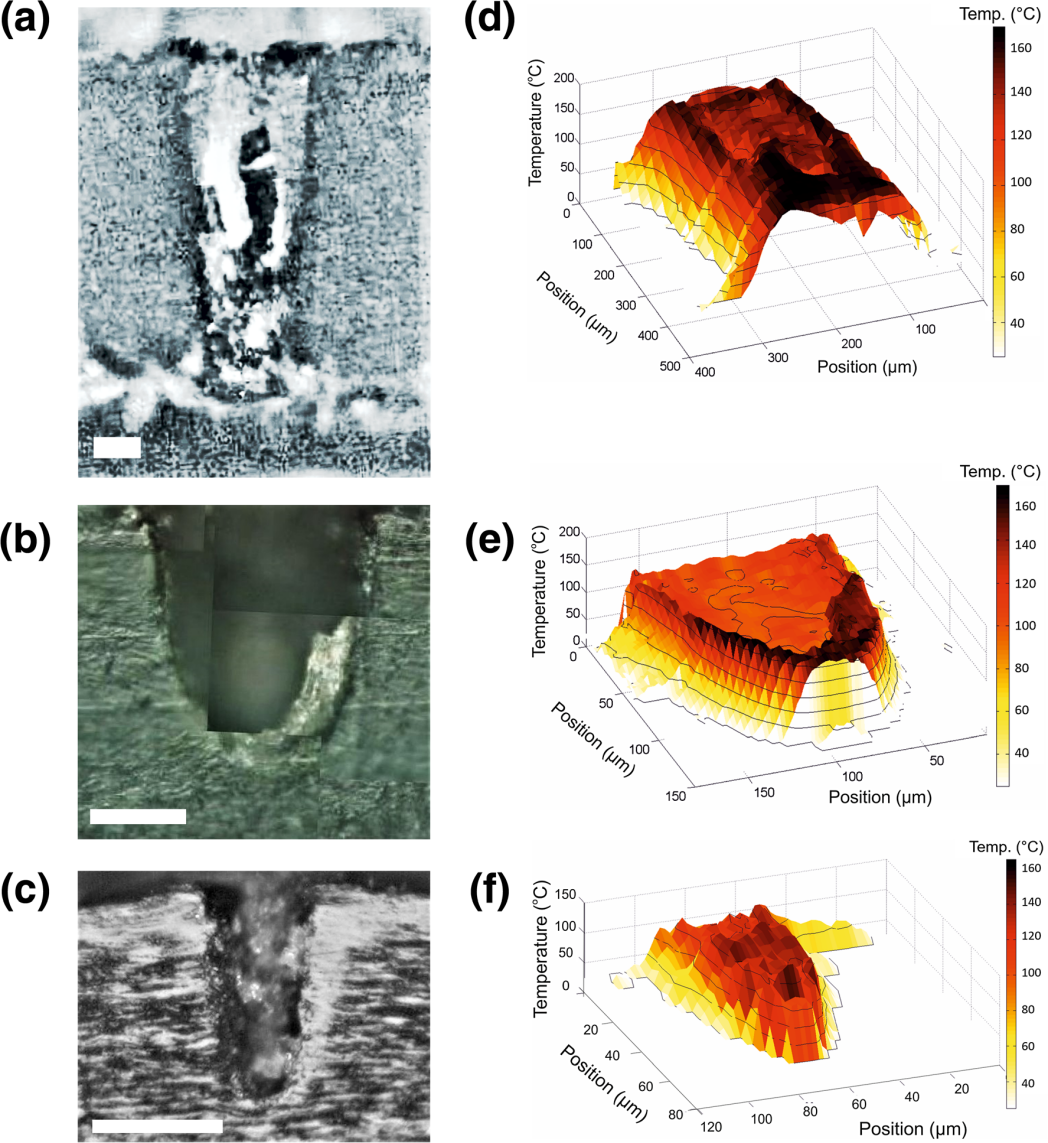
**a**–**c** Optical images of pores created by regimens #1, #3 and #7 (Table 1), respectively; all scale bars shown correspond to 50 μm. The corresponding three-dimensional fluorescence maps are in **d**–**f** providing a quantitative description of the thermal damage surrounding the pores. Contours lines are shown starting at 25 °C and every 25 °C after that. The reader’s attention is drawn to the fact that the scale of the position axis for these panels is not the same

Cross sections of three laser-porated nails using regimens #1, #3 and #7 are shown in Fig. 7a–c, respectively; Fig. 7d–f is the corresponding three-dimensional temperature-position maps converted from fluorescence intensity data. Each pixel in the maps of Fig. 7e and f corresponds to an area of [5 × 2] μm^2^, while that in Fig. 7d represents a 10 × 10 μm^2^ square. The spectra recorded at each pixel were converted to temperatures using Eq. (3) to provide a quantitative ‘heat-map’ of the thermal effects on the nail surrounding the pore. The highest temperatures were detected in the area immediately bordering the pore edge, which appears darker in the optical images, matching that observed in the furnace-heated samples. This area, immediately bordering the pore, also overlaps with that which showed the strongest fluorescence in confocal microscopy. Bright grey areas within the pores in Fig. 7a, b are probably flakes of tissue created during microtoming. Again, data on the temperature maps (Fig. 7d–f) corresponding to the internal regions of the pores cannot be reliably interpreted, because the microscope was out of focus in these areas.

At relatively short distances away from the pores, the nail is unaltered both visually and spectroscopically. For the partially porated samples in Fig. 7e, f, there is a noticeable reduction in the distance to which thermal damage has occurred from the pore edge with increasing pore depth and, below the bottom of the pore, little or no thermal effects are observed. Figure 7d shows thermal damage across the entire pore in the nail.

For the fully porated sample (Fig. 7d), the thermal damage extends approximately 250 μm across the nail. The maximum temperatures achieved in the nail were about 160 °C. For the partially porated samples, the thermal damage decreases with increasing depth into the pore, and the maximum temperatures attained become smaller. For example, the data in Fig. 7e, corresponding to the pore in Fig. 7b, show that thermal damage extends approximately 22 μm at the exterior surface of the nail falling to less than 10 μm at the base of the pore; the corresponding values (Fig. 7f) for the shallower pore (shown in Fig. 7c) are 20 μm and 6 μm, respectively. Fluorescence spectra taken from the nail below the bottom of the partially porated pores were indistinguishable from intact, untreated nails within distances of 6 and 2 μm, suggesting the rapid attenuation of energy penetration.

### Mechanism of poration

Ablation of weakly absorbing biological media with a single femtosecond laser pulse, or with a train of pulses at low repetition rates (≤ 1 kHz), is usually described as a plasma-mediated process initiated by laser-induced breakdown of the tissue [6]. The formation of plasma is accompanied by tissue disintegration/vaporisation. The quasi-free electrons required for this process are produced by an interplay between multi-photon ionisation and avalanche ionisation [6]. The ablation process begins when the irradiance threshold is exceeded. For femtosecond pulses, a much higher irradiance is required for optical breakdown than for longer, nanosecond or microsecond, pulses. It has been calculated that [7], for 200-fs pulses at *λ* = 532 nm, the optical breakdown irradiance threshold is ~ 5 × 10^12^ W/cm^2^, and the radiation exposure threshold is ~ 1 J/cm^2^.

At repetition rates in the MHz range, the ablation mechanism is predicted to be different and due to an accumulative free-electron-mediated chemical decomposition (i.e. bond breaking) in the low-density plasma regime (at irradiances much lower than the optical breakdown threshold) [7]. In the experiments described here, 200-fs laser pulses were delivered via an optical fibre that produced a light spot of 20 μm in diameter on the nail surface. As a result, for nails treated as described in Table 1, poration was performed using pulses with irradiances of (0.12–5.0) × 10^9^ W/cm^2^ and radiation exposures of 0.25–1.3 × 10^−3^ J/cm^2^, i.e. well below the optical breakdown threshold. Plasma-mediated chemical effects in biological media are usually associated with two mechanisms: either decomposition of water into highly reactive oxygen species (OH* and H_2_O_2_), or via a direct chemical change of the organic molecules in resonant electron-molecule scattering [7]. As the water content of nails is very low (3–30%) [37, 38], the latter phenomenon probably dominates in the experiments reported here.

The laser poration process and the thermal damage provoked in the region surrounding the pores can be interpreted as follows. Laser radiation is initially absorbed by the dye on the surface of the nail, confining the energy to a small volume of tissue. Equally, molecules of the dye supply the ‘seed’ electrons required to initiate the avalanche ionisation process. When a critical electron density is reached, a plasma is created and material is ejected from the sample. Dye molecules are ejected too. However, this does not stop the poration process, because damaged biomolecules now become the source of seed electrons. Indeed, rupturing just a single bond in a biopolymeric structure can induce a cascade of bond-breaking events and result in a dramatic lowering of the ablation threshold [7]. As the poration continues, the central region of the base of the pore is always exposed to the highest intensity in the Gaussian-shaped beam, and material is continually removed by plasma ablation. However, the fluorescence maps suggest that a thermal process is causing, or at least contributing to, the removal of material at the pore edges. Laser energy also travels into the tissue adjacent to the pore edge causing an increase in the measured fluorescence response (*I*(*T*,*z*)).

Around the pore drilled with the lowest power (regimen #7), the increased temperature in the tissue indicates that energy has been transferred but has not been sufficient (in terms of power and exposure time) for tissue removal by a photothermal process. In contrast, the pore created with twice the average power (regimen #3) is noticeably wider and larger than expected given the diameter of the fibre. Considering, in order, the three pores shown in Fig. 7a–c as a time-lapse leading to the creation of a fully porated nail, the low level of thermal heating seen around the pore in Fig. 7f may be viewed as the starting point for photothermal ablation. Continued irradiation of this nail would have led to a further increase in temperature, causing ejection of material and enlarging of the pore to its maximum width; the latter, of course, depends on the distance between the fibre and the nail surface and on the numerical aperture of the fibre. Plasma-mediated ablation occurs at the base of the pore, with the energy confined to the volume of material that is ejected, and this explains the minimal thermal damage seen below the pores. Eventually, plasma ablation porates all the way through the nail barrier, and photothermal ablation may keep widening the pore. The increasing ratio of thermal damage to pore width, with increasing depth into the fully porated nail is caused by two physical processes: (a) increase of the beam waist with axial distance, and (b) more light is scattered in the tissue as the beam travels deeper. Both processes contribute to a lower energy density, insufficient for ablation, but large enough to cause thermal damage to the remaining tissue.

In summary, therefore, the results suggest that practically all energy from the central part of the laser beam (which interacts with the nail at the bottom of the pore) is effectively converted into tissue ablation. The incident light is initially strongly absorbed by seed electrons and this leads to a rapid increase in electron density probably via avalanche ionisation processes. As soon as a threshold density of free electrons for plasma formation is reached, a micro-explosion occurs [6] that evaporates and removes a microscopic volume of nail tissue from the pore. As all these processes are extremely fast, very little thermal energy is transferred to the surrounding tissue and so little damage occurs at the bottom of the pore, i.e. at the forefront of the laser beam [7]. Outside this area, the energy from many laser pulses is accumulated and is eventually transferred to the surrounding tissue creating a progressively thicker layer of thermal damage.

**Fig. 8:**
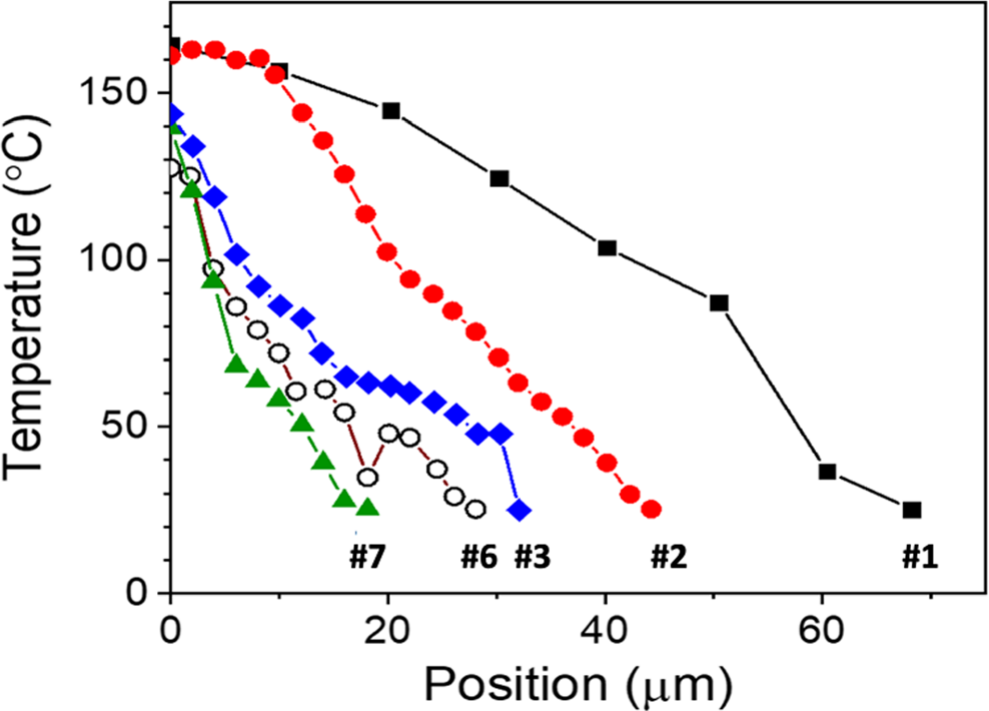
The regions of damage, measured at a depth of 40 μm into the nail, as a function of the radial distance from the edges of five pores created with regimens #1, #2, #3, #6 and #7

Optimisation of the approach, to create pores with minimal damage, requires an understanding of the heat transfer process during poration. Figure 8 shows the thermal damage surrounding four pores, measured radially from the pore edge at a depth of 40 μm. A comparison of the two fully porated samples, one of which was produced without a chopper (regimen #1) and the other with (regimen #2), shows that heat accumulation is significantly larger in the former. Using a chopper producing 2.5-ms pulse trains, separated from each other by 2.5-ms time intervals, makes it possible to significantly reduce thermal damage around the pore. Hence, with regimen #1, thermal damage spreads about 70 μm from the pore edge, whereas, for regimen #2, this distance was reduced ~ 45 μm. The chopper provides breaks in the pulse train and allows the sample to cool down, preventing tissue coagulation and allowing better control over thermal damage around the pore.

Comparison of two partially porated samples (regimens #3 and #7) in Fig. 8 shows that thermal damage also depends on the average power of the laser beam. The pore produced with a power of 0.5 W had thermal damage extending to 28 μm, whereas the corresponding distance from that created with 0.25 W was only 18 μm. It appears, therefore, that confining the energy to a small volume is key to efficient ablation.

Computer simulations of temperature changes in water (which is often used as model system in which to study the interaction of laser irradiation with transparent biological tissue) exposed to a long train of 100-fs pulses at 800 nm with an 80-MHz repetition rate have been reported [7]. Temperature profiles around the laser spot in both radial and axial directions showed a very sharp attenuation, falling by an order of magnitude over distances of just micrometres from the laser focus. The temperature distributions around the pores drilled in human nails are much broader (Fig. 8), a difference believed to be due to the much smaller numerical aperture (0.04) of the optical system used in the experiments reported here compared those employed in the simulations (namely, 1.3 and 0.6). Hence, while a larger numerical aperture makes it easier to confine laser irradiation to a small volume, this is not a suitable strategy for drilling nails as the pores produced are conical rather than cylindrical [4]. The results described here show that, using a hollow-core fibre to deliver femtosecond laser pulses, allows the creation of cylindrical pores with high aspect ratios (e.g. Fig. 7a).

## Conclusions

The potential to improve the clinical treatment of nail disease using laser poration to facilitate drug access to the pharmacological target requires a number of issues to be resolved: (a) control of the depth of nail poration; (b) minimising thermal damage to, and coagulation of, surrounding tissue that may prevent efficient drug diffusion from the pores; (c) creation of small-diameter, cylindrical pores to compromise the nail as little as possible; and (d) ensuring that patient discomfort is avoided.

The research presented here describes the first optical poration of the human nail using a high-repetition-rate femtosecond pulsed laser, from which the light is delivered by a negative curvature photonic crystal fibre. Manipulation of laser power and time of exposure permits small-diameter pores of different depths to be created. Use of an optical chopper to interrupt the train of pulses improves the dissipation of heat from the nail and reduces collateral thermal damage at and near the pore edge.

Fluorescence microspectroscopy was employed as a quantitative tool to assess thermal damage to the nail induced by laser poration. An empirical relation was established to correlate the integrated fluorescent intensity with the increased temperature produced in the nail by exposure to the laser radiation. The high resolution of the Raman microscope permitted detailed maps of thermal damage to be determined and facilitated understanding of the poration mechanism. It was deduced that plasma-driven ablation occurred through the centre of the pore and caused little damage to the nail below. In contrast, cumulative photothermal processes were responsible for removal of material (and the local damage) at the edges of the pore. The results showed that thermal damage progressively decreases with decreasing total energy per pore even when the exposure time for the lower energy is significantly longer. The treatment regimen corresponding to the lowest energy per pore caused minimal thermal damage.

While the method developed offers a useful tool for the quantitative evaluation of thermal damage to the human nail, it should also be applicable to other, diverse biological tissues, such as skin, bone and teeth. Furthermore, it appears that the approach is sufficiently robust to take into account the position-dependent variation in optical properties of inhomogeneous biological material as well.
